# Supplementary material for: Slice-PASEF: Maximising Ion Utilisation in LC-MS Proteomics
Source: bioRxiv. 2025 Sep 2:2022.10.31.514544. Preprint. [Version 2] doi: 10.1101/2022.10.31.514544 (PMC12424990; doi:10.1101/2022.10.31.514544)
Supplement: Supplement 1 [file media-1.pdf]

Supplementary Materials for

Slice-PASEF: maximising ion utilisation in LC-MS proteomics

Ludwig R. Sinn[1,❋,✉], Lukasz Szyrwiel[1,❋], Justus Grossmann[1], Kate Lau[1], Katharina Faisst[1], Di Qin[2,3], Florian Mutschler[4,5], Luke Khoury[6], Andrew Leduc[6], Markus Ralser[1], Fabian Coscia[2], Matthias Selbach[4], Nikolai Slavov[6], Nagarjuna Nagaraj[7], Martin Steger[8] and Vadim Demichev[1,✉]

[1]Department of Biochemistry, Charité – Universitätsmedizin Berlin, Berlin, Germany
[2]Spatial Proteomics Group, Max-Delbrück-Center for Molecular Medicine in the Helmholtz Association, Berlin, Germany
[3]Charité – Universitätsmedizin Berlin
[4]Proteome Dynamics, Max Delbrück Center for Molecular Medicine in the Helmholtz Association (MDC), Berlin, Germany
[5]Faculty of Life Sciences, Humboldt-Universität zu Berlin, Berlin Germany
[6]Departments of Bioengineering, Biology, Chemistry and Chemical Biology, Single Cell Proteomics Center and Barnett Institute, Northeastern University, Boston, MA, USA; Parallel Squared Technology Institute, Watertown, MA, USA
[7]Evotec International GmbH, Neuried, Germany
[8]NEOsphere Biotechnologies GmbH, Planegg, Germany
❋These authors contributed equally
✉Correspondence to: ludwig-roman.sinn@charite.de, vadim.demichev@gmail.com

This file includes:

Figure S1: Distribution of precursor m/z along ion mobility slices

Figure S2: Quantitative similarity between dia-PASEF and 1-Frame Slice-PASEF methods on analytical flow rate LC-MS

Figure S3: Figures of merit for dia-PASEF and 1-Frame Slice-PASEF methods comparison on U2OS single-cell proteomics

Figure S4: Overall identifications in plexDIA Slice-PASEF and their relationship to cell size and quantitative precision

Figure S5: Differential protein abundances in biological processes characteristic to Jurkat and U937 cell lines

Figure S6: Quantitative protein abundance differences between NUCKS1-stratified groups in U937 cells

Figure S7: Proteomics and Ubiquitinomics Identifications upon drug treatment

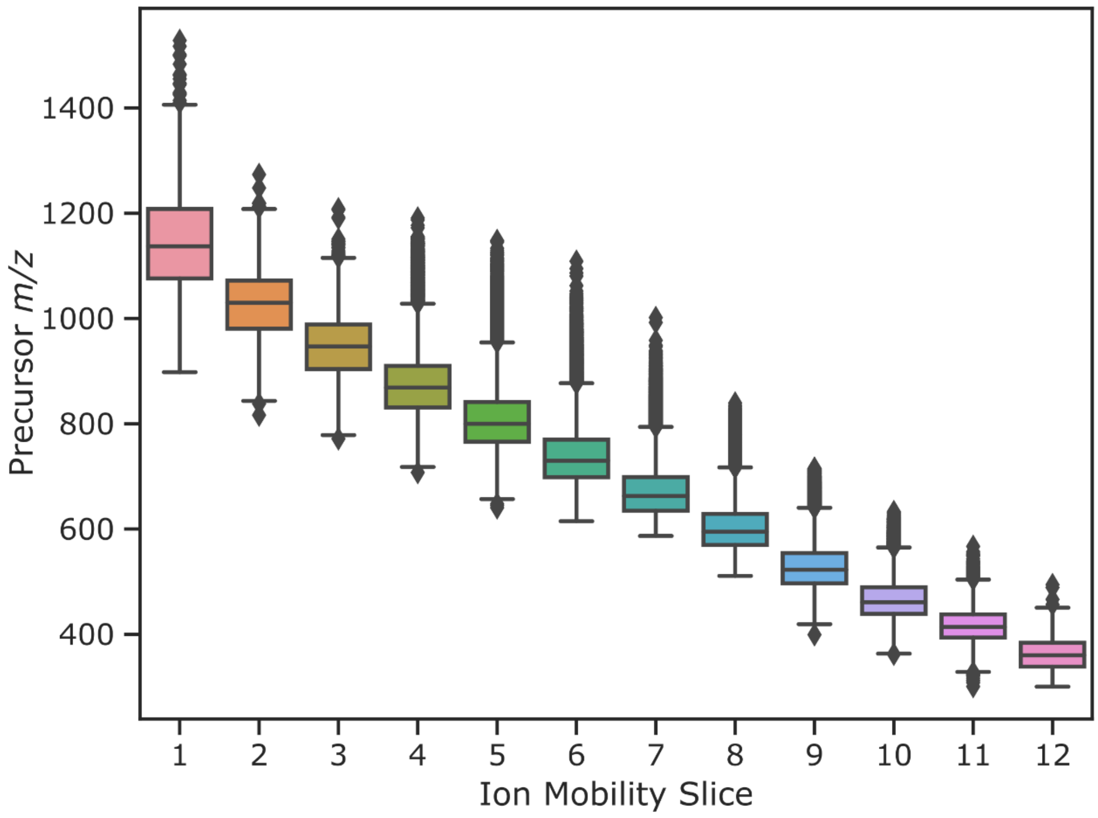

Fig. S1: Distribution of precursor m/z along ion mobility slices

Boxplots on precursor ion distributions in dia-PASEF data from Meier and coworkers13. Data was filtered to the ion mobility range of interest (m/z 350-1050 & 0.75-1.15 V*s/cm2) and split into twelve bins using 0.0375 V*s/cm2 intervals. The interquartile range (IQR) (i.e., 25-75% of data) equals 71.18 m/z (±24.48 s.d.) and decreases along the slices (towards lower ion mobility). Whiskers span over 1.5 x IQR. Outliers are shown as black diamonds.

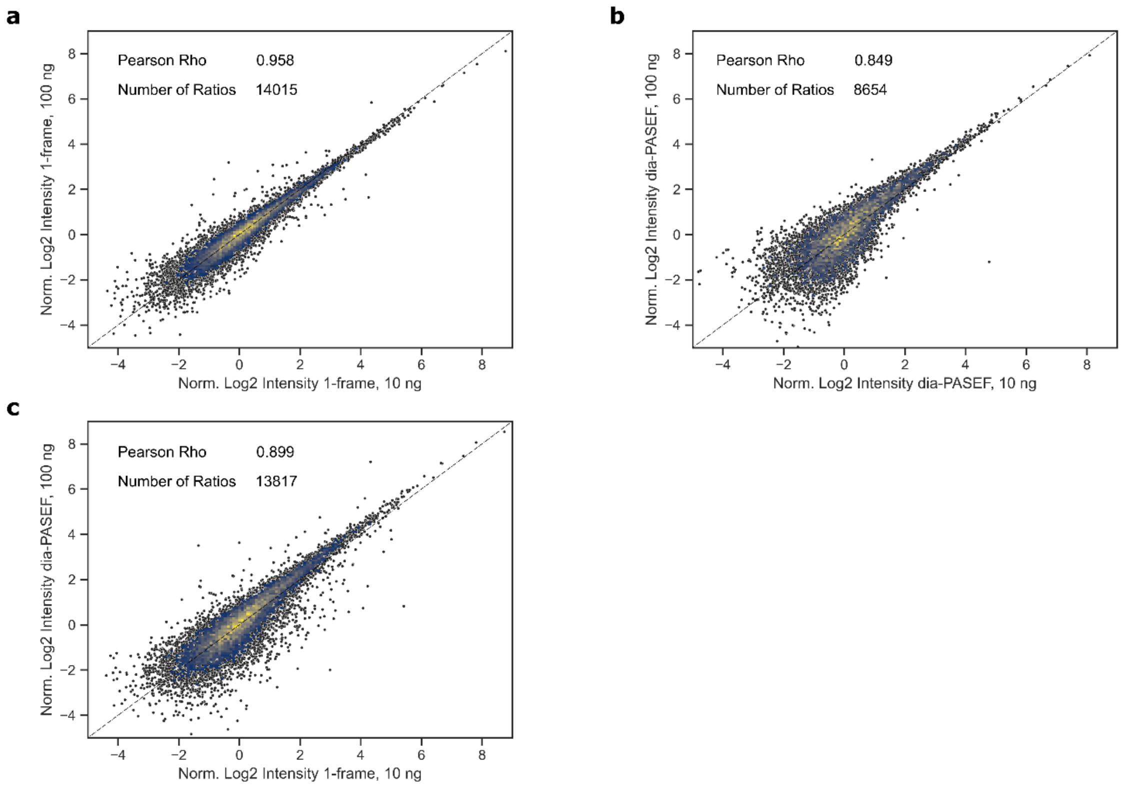

Fig. S2: Quantitative similarity between dia-PASEF and 1-Frame Slice-PASEF methods on analytical flow rate LC-MS

Normalised log_2-transformed signal intensities of shared precursor identifications are shown for different method combinations and injection amounts. a) Comparison of 10 and 100 ng K562 loads using 1F Slice-PASEF data. b) Comparison of 10 and 100 ng K562 loads using dia-PASEF data. c). Comparison of 100 ng dia-PASEF vs. 10 ng 1F Slice-PASEF data, both processed in independent DIA-NN searches. Input quantities represent the median from three technical replicates.

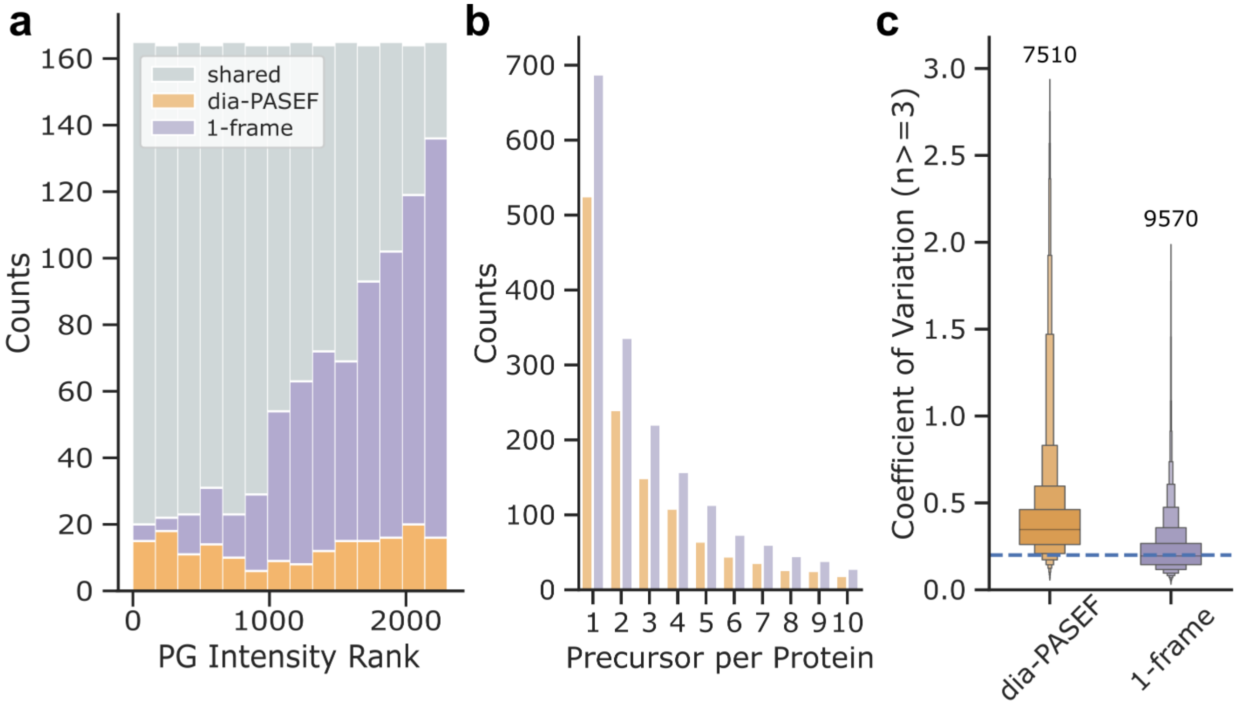

Fig. S3: Figures of merit for dia-PASEF and 1-Frame Slice-PASEF methods comparison on U2OS single-cell proteomics

a) Proteome coverage by intensity-binned protein groups from both methods. Identifications of dia-PASEF are shown in orange, 1-Frame Slice-PASEF in purple, and shared identifications in grey. b) Average identified precursors per protein for both methods. c) Quantitative precision for identified precursors of both methods as Boxenplot. Medians (dia-PASEF: 0.347 / 1-Frame Slice-PASEF: 0.195) and 50% IQRs are highlighted by the central box while each quantile further outwards represents half of the remaining fraction. Outliers (i.e. upper and lower 0.7%) are not shown. The sample size for each method is given above the boxes. The dashed line marks a CV of 20%.

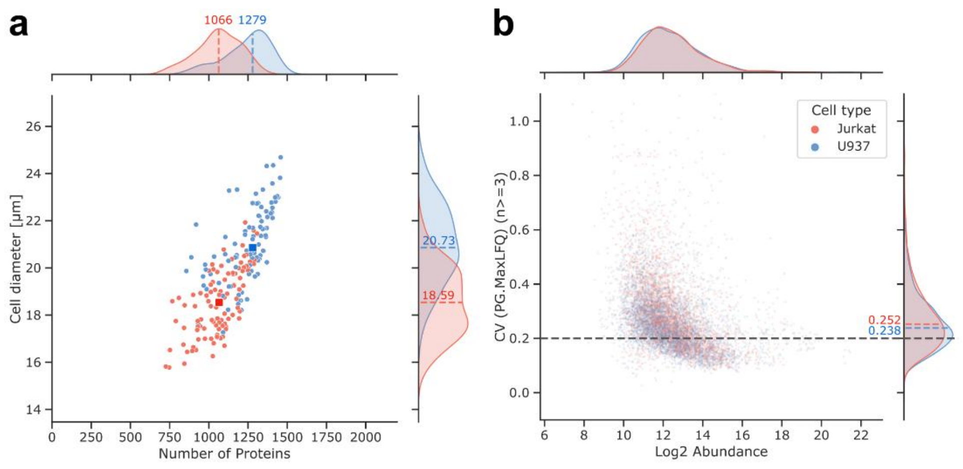

Fig. S4: Overall identifications in plexDIA Slice-PASEF and their relationship to cell size and quantitative precision

a) The number of identified protein groups increases with cell size. Medians per cell type are highlighted by a darker rectangle for each cell type with the corresponding values and distributions being indicated at the axis margins. b) Quantitative precision measured by the coefficient of variation (CV) per protein group across the quantitative domain. The dashed black line marks a CV of 20% while the dashed lines at the right axis margin mark the medians for each cell type (Jurkat: 25.2% / U937: 23.8%). The colour code for each cell type is indicated in panel (b).

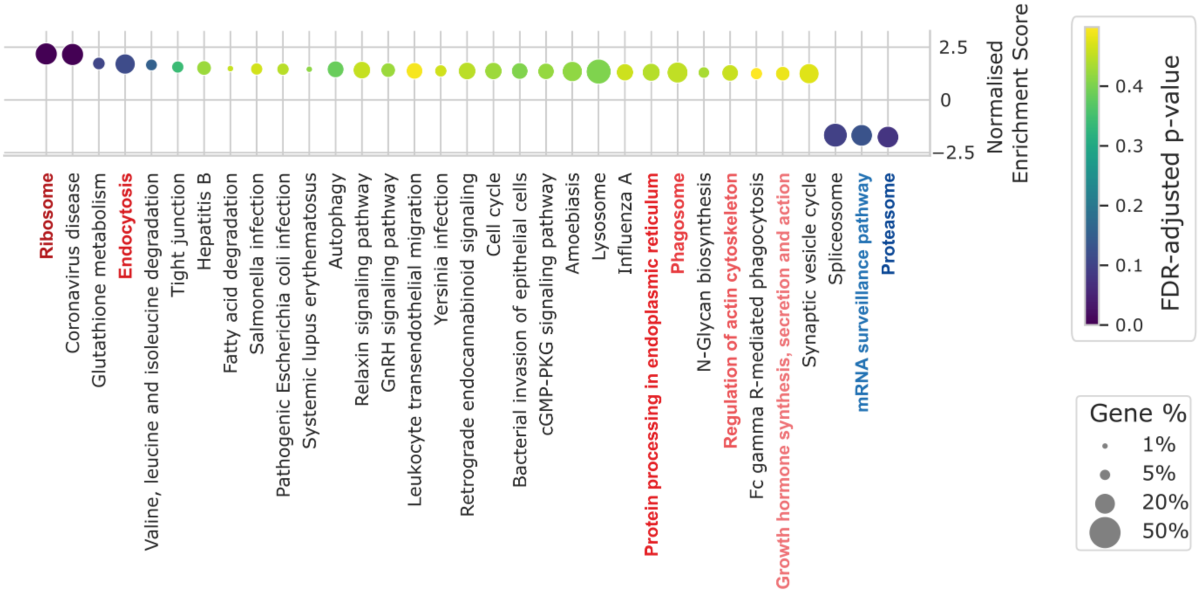

Fig. S5: Differential protein abundances in biological processes characteristic to Jurkat and U937 cell lines

Gene set enrichment analysis (GSEA) on quantitatively different shared gene products of both cell types. Upon applying a 10% completeness filter, gene products were ranked by a score derived from Mann-Whitney-U p-values and the direction of fold-change (Methods) before forwarding to pre-ranked GSEA. Terms passing a 50% FDR q-value threshold are displayed with declining normalised enrichment scores. The colour hue illustrates multiple testing-adjusted p-values while the size of each sign symbolizes the proportion of genes at the enrichment score peak as defined by the GSEApy Python package. Selected gene sets are highlighted in red for U937 and in blue for Jurkat cells.

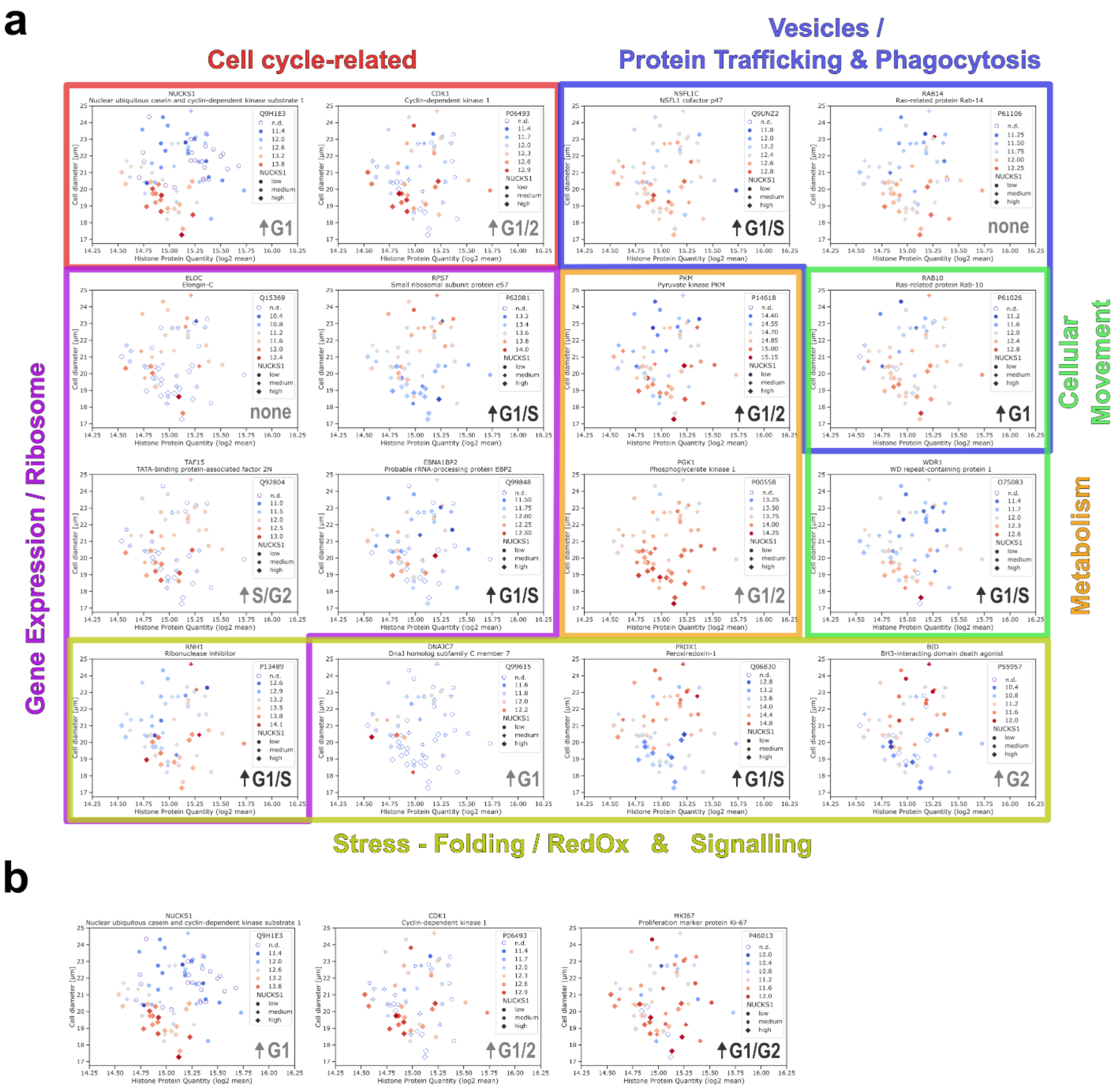

Fig. S6: Quantitative protein abundance differences between NUCKS1-stratified groups in U937 cells

The log2-converted, normalised and averaged quantities of histone proteins are compared with observed cell sizes, separated into abundance tertiles of the NUCKS1 gene product (not detected, n.d.: hollow circle, low: circle, medium: cross, high: diamond). The colour hue illustrates the corresponding quantities of a protein of interest, as indicated at the top right of each subpanel. Panel (a) shows the significant hits (10% FDR cut-off, from performing a Student's t-test and Benjamini-Hochberg multiple testing correction) from comparing the proteins between the low and high NUCKS1 abundance tertiles. Proteins were manually associated with broad biological categories, as indicated by border and label in the same colour. Suggested cell-cycle stages associated with higher levels of each gene product were inferred from data by Altun and colleagues41 and are highlighted in black if ≤10% FDR therein. Panel (b) compares NUCKS1

with two cell-cycle marker proteins, CDK1 (also present in panel a) and MKI67, previously highlighted as cell cycle markers by Bubis and colleagues39. Data were processed as for panel (a).

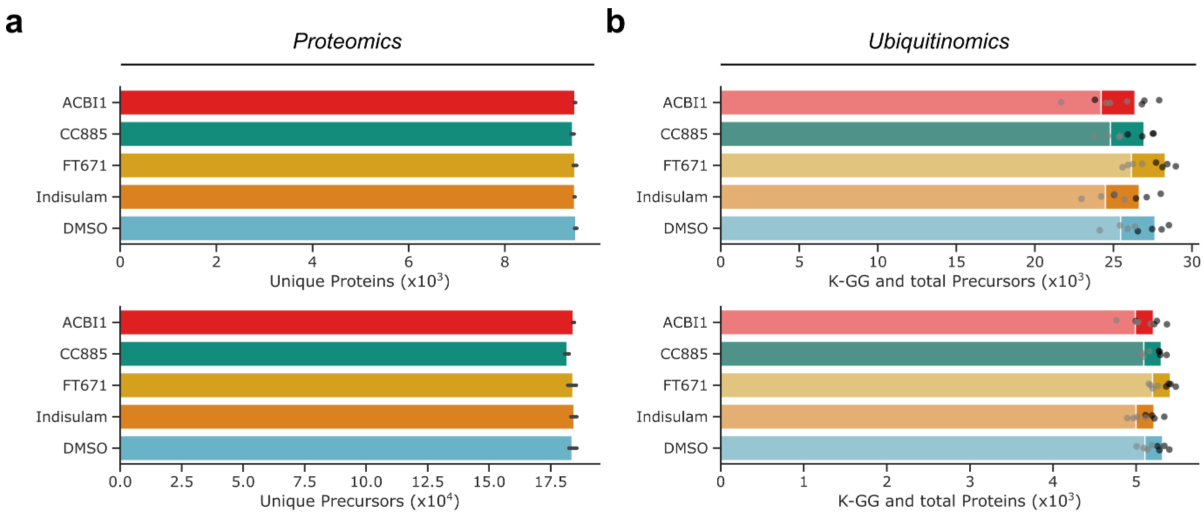

Fig. S7: Proteomics and Ubiquitinomics Identifications upon drug treatment

a) Protein group and precursor identification numbers in drug treatment experiment using dia-PASEF proteomics. Error bars span the 95% confidence interval. b) K-GG remnant peptide precursors and ubiquitylated protein group identification numbers in Ubiquitinomics drug treatment experiment using 1-Frame Slice-PASEF. Lighter colours symbolize K-GG-remnant entities while darker colours indicate unmodified precursor or protein group identifications. Individual values are shown as jittered points. DMSO represents the mock treatment.
